# The mesocorticolimbic system in stimulant use disorder

**DOI:** 10.1038/s41380-025-03148-0

**Published:** 2025-09-10

**Authors:** Edythe D. London, Stephanie M. Groman, Marco Leyton, Harriet de Wit

**Affiliations:** 1 Department of Psychiatry and Biobehavioral Sciences, Department of Molecular and Medical Pharmacology, and the Brain Research Institute, University of California, Los Angeles, CA USA; 2 Department of Anesthesia and Critical Care, University of Chicago, Chicago, IL USA; 3 Department of Psychiatry, McGill University, Montreal, QC Canada; 4 Department of Psychiatry and Behavioral Neuroscience, University of Chicago, Chicago, IL USA

**Keywords:** Neuroscience, Psychology

## Abstract

Stimulant Use Disorder (StUD) is a pervasive and extremely dangerous form of addiction for which there are currently no approved medications. Discovering treatments will require a deep understanding of the neural mechanisms underlying the behavioral effects of stimulant drugs. A major target is the mesocorticolimbic system. Individual differences in mesocorticolimbic function can influence the propensity to initiate stimulant use and the risk for stimulant use disorders. Since repeated stimulant use can further alter mesocorticolimbic function, these pathways may serve as a target for both early interventions aimed at preventing the onset of harmful stimulant use and treatments designed to alleviate addiction symptoms. Here we review evidence from studies in both humans and laboratory animals, focusing on the neurotransmitter systems most strongly implicated in StUD, primarily dopamine and, to a lesser extent, glutamate. We identify evidence of (i) complex, non-linear perturbations to mesocorticolimbic function related to stimulant use, and (ii) gaps in knowledge and opportunities for research to improve our understanding of the determinants and consequences of StUD.

## Introduction

The use of cocaine and amphetamine-type stimulants has increased globally during the past decade, aggravated by the appearance of new and more potent synthetic stimulant drugs [1, 2]. Together, these stimulants now contribute to almost 50% of overdose deaths, accelerating a fourth wave of the overdose epidemic [3, 4]. Despite this, no medications have been approved for the treatment of Stimulant Use Disorder (StUD), and behavioral treatments have limited availability and only modest success [5]. A deeper understanding of the mechanisms driving the initiation and persistence of stimulant use is critical for developing more effective interventions. Emerging evidence points to neurochemical processes and neural circuitry within the mesocorticolimbic (MCL) system as well as the behaviors they modulate.

This review begins by delineating the neuroanatomical and neurochemical architecture of the MCL system. We then explore how features of MCL circuitry contribute to the initiation of stimulant drug use and how individual variability in the initial drug response can influence the likelihood of repeated use. Next, we synthesize current evidence implicating MCL dysfunction in the behavioral phenotypes associated with StUD. Finally, we identify critical gaps in the existing literature and propose that a mechanistic understanding of MCL function in StUD can guide the development of novel therapeutic interventions. While data on newer stimulants, such as synthetic cathinones (‘bath salts’) have been reported [6], this review focuses on the more prevalent substances: cocaine and amphetamine-type stimulants.

## What defines the MCL?

**Fig. 1:**
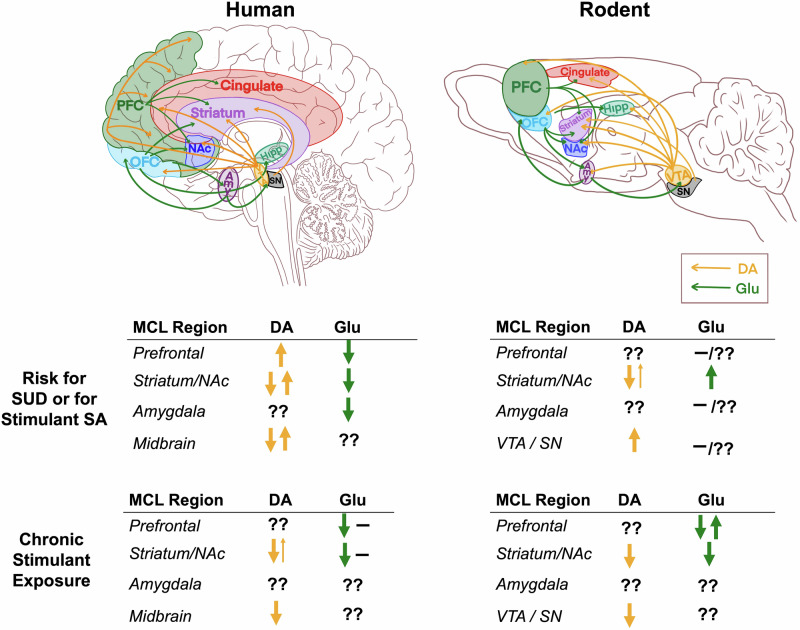
Schematic of MCL regions involved in risk for, and consequences of, stimulant exposure in humans and rodents. Ascending dopaminergic projections from midbrain nuclei innervate the cerebral cortex, basal ganglia, and limbic structures. In parallel, descending glutamatergic pathways innervate subcortical limbic regions, which in turn project back to the midbrain, forming a reciprocal circuit. The table summarizes key findings from the literature and gaps in knowledge linking markers of dopaminergic and glutamatergic activity to risk for substance use disorders in humans and to stimulant self-administration in rodents, as well as alterations observed following chronic stimulant exposure. Amy amygdala, DA dopamine, Glu glutamate, Hipp hippocampus, NAc nucleus accumbens, OFC orbitofrontal cortex, PFC prefrontal cortex, SA self-administration, SN substantia nigra, SUD substance use disorder, VTA ventral tegmental area. Arrows up or down signify higher or lower levels; the appearance of both up and down arrows indicates reports of both effects; ―, no group difference; ??, limited or no evidence.

The mesocorticolimbic (MCL) system includes connections between the upper brainstem—such as the ventral tegmental area (VTA), substantia nigra (SN), raphe nuclei, and locus coeruleus—and striatal, limbic (e.g., amygdala, hippocampus), and cortical structures (e.g., orbitofrontal cortex [OFC], medial prefrontal cortex [mPFC], anterior cingulate cortex [ACC]) [7, 8]. Dopaminergic, noradrenergic, and serotonergic projections ascend from the brainstem to the cerebral cortex, basal ganglia, and limbic structures. In turn, descending glutamatergic corticostriatal projections provide excitatory input to the VTA, SN, and subcortical limbic regions, which send reciprocal projections back to the cortex and striatum (see Fig. 1 depicting dopaminergic and glutamatergic pathways).

These projections create topographical loops that appear to segregate according to function. For example, cortical areas involved in motor function send projections to the dorsal striatum, which projects to the globus pallidus and the SN before returning to the cortex. Cortical areas involved in reward processing (e.g., OFC) receive dense innervation from VTA dopamine neurons and send descending projections to the ventral striatum, amygdala, and hippocampus, each of which also projects directly or indirectly back to the dopamine cell bodies in the midbrain. Repeated stimulant exposure induces progressive neuroadaptations within key cortico-striatal and limbic circuits, which are hypothesized to underlie the development of StUD and contribute to dysregulation of reward processing, impulse control, and decision-making.

Given the structural and functional interconnectivity of the MCL system, we propose that abnormalities in any individual brain region within this network may reflect broader disruptions of the MCL system, regardless of whether they result from altered midbrain transmission. This perspective is supported by evidence that dysfunction in isolated MCL components (e.g., midbrain, striatum, or prefrontal cortex) can produce similar behavioral outcomes. This approach contrasts with more conservative models, which contend that evidence of dysfunction across multiple MCL regions is necessary to infer MCL system alterations.

This review focuses on dopaminergic and glutamatergic transmission, both of which are implicated in the emergence and persistence of StUD. Dopamine and glutamate play complementary roles―dopamine drives the acute responses to reward and glutamate mediates long-term synaptic plasticity and mechanisms of relapse [9–11]. Other neurotransmitters, including serotonin and norepinephrine, provide modulating influences within the MCL, and their role in addiction has been reviewed in detail elsewhere [12–14].

## How is the MCL involved in vulnerability to StUD?

### Human studies

#### Neurochemistry (Fig. 1)

Several lines of evidence suggest that individual differences in MCL dopamine transmission affect risk for initiating substance use. Large genome-wide association studies have implicated genetic variants related to dopamine regulation in SUDs across several pharmacological classes [15]. Additional evidence for dopamine involvement comes from observations that neurological patients treated with D3 receptor (D3R) agonists frequently develop addiction-like behaviors [16]. Positron emission tomography (PET) studies in humans have shown that stimulant drugs enhance dopaminergic activity, with individual differences in this response associated with the subjective and behavioral effects of the drugs [17–19].

Moreover, SUD-associated traits, such as impulsivity and novelty-seeking, have been linked to lower availability of D2-type dopamine receptors (D2/3R, encompassing D2 and D3 subtypes), as measured by binding potential in the midbrain [20], where most D2/3Rs function as autoreceptors, and to greater amphetamine-induced striatal dopamine release [21–24]. Conversely, higher D2/3R availability in both striatal and extrastriatal regions has been observed in individuals at high risk for SUD [25–29]. Higher striatal D2/3R availability also has been observed in unaffected individuals who are family history-positive for SUD compared to family history-negative controls, raising the possibility that elevated D2/3R availability acts as a protective factor [30]. Far fewer studies have investigated glutamate in humans, but one study found lower MCL metabotropic glutamate receptor type 5 (mGlu5) availability in youths at greater risk for SUD compared to low-risk individuals [31]. This finding, however, was complicated by early use of cannabis in the high-risk youths.

Compelling evidence indicates that stimulant use can lead to long-lasting changes in dopamine-related features [32]. In humans, repeated administration of amphetamine can lead to greater striatal dopamine release in response to the drug (i.e., sensitization) and to cues paired with the drug (i.e., conditioning). Sensitization is more pronounced in more impulsive participants [32, 33], and conditioned dopamine responses are greater in those who report stronger amphetamine-induced positive effects [34]. In addition, greater lifetime histories of stimulant drug use were associated with larger striatal dopamine responses to cocaine in nondependent individuals [35]. Conditioned dopamine responses might also vary with the extent of past use. Following three amphetamine doses, conditioned dopamine responses can be seen in the ventral striatum [34]. In comparison, individuals with a history of more extensive stimulant use exhibit these effects in the dorsal striatum [36], a region associated with the development and persistence of extinction-resistant, habit-like behaviors [37].

Collectively, these findings suggest that a combination of inherent differences in dopamine function and alterations induced by ‘recreational’ drug use may influence the risk of developing substance use disorders (SUDs). However, the nature of this relationship remains uncertain, as both heightened and blunted dopaminergic responses might contribute to vulnerability. Further evidence on variations in dopaminergic responses to initial doses of stimulants may provide a biomarker for future repeated use, albeit clinically impractical.

#### Morphometry/connectivity/reactivity

Neuroimaging studies suggest that morphological and functional features of the MCL system may reflect inherited risk for StUD. While these studies generally do not directly assess dopaminergic projections, they consistently report abnormalities within or between key MCL target regions. For example, both individuals with stimulant dependence and their unaffected siblings exhibit greater gray-matter volume in the putamen and amygdala, along with reduced fractional anisotropy―indicating a deficit in structural connectivity of the inferior prefrontal cortex (PFC) compared of healthy controls―suggesting disrupted structural connectivity [38]. Such findings indicate that neurobiological markers of StUD risk may be present even in unaffected individuals with family loading [38]. Moreover, individuals with a family history of SUDs show weaker functional connectivity between the ventromedial caudate, left orbitofrontal cortex (OFC), and ventromedial PFC [39]. In contrast, a subset of high-risk individuals—primarily male—who did not develop StUD demonstrated stronger connectivity in networks implicated in top-down inhibitory control (e.g., between the right lateral PFC and the medial caudate nucleus) and habit regulation (e.g., supplementary motor area, superior medial frontal cortex and putamen) [39]. Together, these finding suggest that heritable variation in MCL circuitry may confer either vulnerability or resilience to StUD.

In addition to these family studies, a longitudinal study with an fMRI component examined neural responses to the Risky Gains Task in occasional stimulant users who either did or did not transition to problematic drug use [40]. Those who developed stimulant use problems differed from their resilient counterparts in several ways: 1) they made riskier decisions after receiving feedback of winning; 2) they exhibited lower frontal, insular, and striatal activations after win/loss feedback following risky decisions; and 3) they displayed lower thalamic but greater temporo-occipital neural responses to risky losses than to risky wins. The authors suggested that blunted frontostriatal responses to signals of risky outcomes might indicate risk for a future StUD.

Some fMRI studies have tested whether variations in MCL responses during a monetary reward task predicted euphoria from a single dose of amphetamine. One study assessed the relationship between neural activity during anticipation of winning money (while drug-free) and self-reported liking of d-amphetamine (20 mg, p.o.), which was assessed during a separate session [41]. Amphetamine liking was correlated positively with activation in the amygdala but negatively with activation in the insula and caudate during anticipation of monetary reward. In the same study, participants who reported more amphetamine liking also exhibited greater activation in mesolimbic regions, including in the bilateral caudate and putamen, during receipt of monetary reward [42]. Taken together, these findings suggest that individual differences in how non-drug rewards are processed—whether during anticipation or receipt—can predict the pleasurable subjective effects, or ‘liking,’ of amphetamine, potentially serving as a marker for future problematic stimulant use.

Other evidence suggests that future stimulant use is related to neural and behavioral responses during a behavioral inhibition task. In a study of healthy volunteers, the rewarding effects of amphetamine were related to both the euphorigenic effect of amphetamine and future stimulant misuse [43]. Participants completed the Stop-signal Task during fMRI in a drug-free state and then received amphetamine or placebo in a separate session. Neural responses during inhibition (i.e., activation in right prefrontal regions) were negatively correlated with amphetamine-induced euphoria and stimulation. Those who exhibited poorer behavioral inhibition during the task reported greater amphetamine-induced euphoria. These findings are consistent with evidence that neural responses in children performing an inhibitory task predict later substance use [44]. Thus, poor inhibitory control and high sensitivity to amphetamine reward were related at both behavioral and neural levels.

In sum, there are now several lines of evidence suggesting that MCL circuitry differences affect risk for StUD. These include both behavioral and MCL function measures in longitudinal research and studies of non-affected relatives of those with StUD. Individual differences in amphetamine liking might be related to varying MCL responses to non-drug rewards and during a behavioral inhibition task.

#### Behavior

Novelty-seeking, sensation-seeking and impulsivity-related personality traits have been evaluated as potential risk factors for SUD in general [45–47] and for StUD specifically. In one large longitudinal study, high novelty seeking during adolescence predicted any use or frequent use of stimulants during adulthood [48]. Another study found higher levels of sensation-seeking and impulsivity among stimulant-dependent adults when compared with both their non-drug-using siblings and controls [49]. The siblings, however, reported higher levels of impulsivity than controls, but similar levels of sensation seeking, compared to controls [49]. One interpretation is that novelty- and sensation-seeking predispose an individual to initial stimulant use whereas dysregulated impulse-control may increase the risk for transitioning to StUD [28, 38]. In addition, a comprehensive review of personality as a risk factor for SUD found that individuals with low positive emotionality, high negative emotionality, and low constraint are likely to be at greatest risk for SUD, and that the converse of these traits would be protective [50]. Consistent with this perspective, there is also evidence that externalizing behavioral traits in youth—marked by outwardly directed emotions and often expressed through disruptive or impulsive actions—are linked to a heightened risk of early substance misuse and the later development of substance use disorders (SUDs) [51–53]. Although these latter observations concerned SUDs in general, the findings have implications for StUD.

Important knowledge about risk of StUD can also be gained from studying the acute effects of stimulants during the early stages of use. At a behavioral level, initial positive subjective effects of drugs, including stimulants, are thought to predict repeated use. Pleasurable subjective effects from a single, moderate dose of amphetamine predict behavioral choice of the drug over a placebo [54], and repeated use can lead to escalation of use [55, 56]. Indeed, regulatory agencies use the positive subjective effects of new drugs as a key predictor of their potential for misuse [57].

Individual differences in subjective reward valuation—and consequently, drug-taking behavior—may arise from variability in drug effects on the mesocorticolimbic (MCL) system. As noted above, interindividual differences in neural responses are associated with both subjective experiences and behavioral outcomes [17–19]. For example, in healthy adults, methamphetamine increases functional connectivity between putamen and left inferior frontal gyrus (IFG), and this increase correlates negatively with methamphetamine-induced euphoria and stimulation [58].

As indicated in studies with methylphenidate, both behavioral and neural responses to acute stimulant administration depend on a variety of factors, including baseline dopamine receptor availability, sex, rate of onset of the drug effect, expectations, and chronotype (“morningness” vs. “eveningness”) [59–63]. The positive subjective effects of single doses of stimulants have also been linked to personality traits, especially those that are related to dopamine function. For example, reward sensitivity [64, 65], impulsivity [65], physical fearlessness [65], and sensation seeking [66] predicted subjective amphetamine-induced euphoria in healthy volunteers.

In sum, there is evidence that personality traits and cognitive functions thought to involve the MCL influence risk for stimulant use. Behavioral responses to initial doses of stimulant drugs also reveal important variability in MCL function that can predict future drug use.

### Animal studies

#### Neurochemistry (Fig. 1)

##### Dopamine

The first studies implicating dopamine in drug reward found that pimozide—an antipsychotic and D2/3 receptor antagonist—decreased lever pressing for amphetamine and cocaine in a dose-dependent manner [67, 68]. Subsequent work in non-human primates (NHPs) using positron emission tomography (PET) found that dominant male monkeys with higher striatal D2/3R availability compared to their subordinate counterparts at baseline self-administered significantly less cocaine, suggesting that low D2/3R may be a risk factor for susceptibility for drug use [69]. Indeed, individual differences in striatal D2/3R availability in male NHPs has been found to be predictive of rates of cocaine self-administration [70], and viral-mediated overexpression of striatal D2R decreases cocaine self-administration in rats [71]. These data suggest that low levels of striatal D2/3Rs may contribute in part to the development of SUDs in humans. More recent studies, however, have shown that higher D2/3R availability in female NHPs predicts higher rates of cocaine self-administration [72, 73], suggesting that the relationship between striatal D2/3Rs and cocaine self-administration may differ by sex. Although the mechanism for these differences is not known, it may reflect sex differences in striatal dopamine receptor expression [74] and/or in the regulation of dopamine release [75]. Moreover, differences in study design and/or methodology between the male and female NHP studies may have contributed to these divergent findings. For example, males that were susceptible to cocaine self-administration had subordinate status within their social hierarchy and were exposed to repeated attacks [69], whereas females that rapidly acquired cocaine self-administration had dominant status within their hierarchy [73]).

Midbrain dopamine neurons project to the striatum, and evidence suggests that increased midbrain dopamine activity is associated with heightened reward-related behaviors. For example, enhancing the activity of VTA dopamine neurons that project to the nucleus accumbens increases cocaine reinforcement, as measured by a conditioned place preference paradigm, in both male and female mice; however, this effect varies with the estrous cycle in females [76]. Further, phasic release of dopamine in the nucleus accumbens precedes the initiation of drug-seeking behaviors in rats trained to self-administer cocaine [77]. Susceptibility to StUD may therefore reflect a hyperdopaminergic state. Supporting this idea, studies using viral and genetic tools to reduce D2 autoreceptor expression in the VTA—thereby mimicking a hyperdopaminergic state—have shown that such manipulations increase cocaine-taking and cocaine-seeking behaviors in rats and mice [78, 79]. These data suggest that dysregulation in the mechanisms controlling dopaminergic release, such as autoreceptor activation, may contribute to the emergence of StUD. Dopamine release is not solely regulated by D2Rs; emerging research suggests that midbrain D3Rs also play a crucial role in modulating striatal dopamine tone [80, 81] and influencing the propensity for self-administration [82]. Dysregulation of midbrain D2 or D3 autoreceptors may, therefore, be a risk factor for the development of StUDs.

Collectively, these studies in laboratory animals provide compelling evidence for involvement of dopamine in drug reward, drug consumption, and likely susceptibility to SUDs. Nonetheless, further research is needed to delineate how specific disruptions in dopamine signaling modulate drug-taking behavior. The influence of dopamine and D2/3R signaling on susceptibility to drug misuse likely varies by brain region, neural circuits, receptor location (pre- vs. postsynaptic), and subtype [83].

##### Glutamate

Research on stimulant self-administration has yielded mixed findings about the role of glutamate in vulnerability to StUD. Two separate PET imaging studies found that pre-existing differences in mGlu5 availability did not predict cocaine-taking behaviors in rodents [84, 85]. However, mice selectively bred to drink high levels of methamphetamine exhibited greater extracellular glutamate and mGlu5 levels in the nucleus accumbens [86]. These discrepancies may reflect differences between the mechanisms of action of cocaine and methamphetamine (i.e., blocking dopamine reuptake vs. promoting release) and/or a limitation of PET imaging. The use of both in vivo and ex vivo approaches to measure glutamate and mGlu5 may help resolve this apparent discrepancy. Additional research is needed to understand how glutamate receptor subtypes beyond mGlu5 contribute to stimulant drug use or susceptibility to StUD.

#### Morphometry/connectivity

Although abnormalities in MCL structure or function appear to predict addiction liability, including StUD, in humans [38], preclinical evidence supporting this relationship remains limited. This discrepancy may reflect the lack of high-resolution imaging tools and the large sample sizes required for rodent studies (e.g., >30 subjects). Nonetheless, variation in functional connectivity between the PFC and the dorsomedial striatum (DMS) predicts whether a rat will develop compulsive-like cocaine-seeking behaviors. Specifically, lower PFC-DMS connectivity prior to any drug exposure is associated with greater resistance to punishment for cocaine-seeking behaviors, suggesting that impaired prefrontal control over the striatum may promote the transition from occasional to more harmful drug use [87]. The mechanisms underlying hypofunction in this prefrontal-striatal circuit remain unclear but may involve disruptions in maturation of prefrontal circuits during adolescence [88].

#### Behavior

##### Sensitivity to reward

Differences in the response to a reward at the neural and behavioral levels are likely to impact susceptibility to drug use. Rats with greater locomotor responses in a novel environment, which might reflect their reaction to the ‘reward’ of novelty, have a higher propensity to acquire amphetamine and cocaine self-administration [89, 90]. High responders to novelty also exhibit greater cocaine-induced locomotion and nucleus accumbens dopamine responses [91], greater susceptibility to low-dose cocaine-induced conditioned place preferences [92], and higher VTA dopamine cell basal firing rates and bursting activity [89]. Together, these features may influence responsivity to stress and the amount of drug intake [93, 94]. They are less thoroughly studied in NHPs, but a recent study found that the propensity to value an appetitive reward (e.g., sweetened condensed milk solution) was associated with morphological differences in the MCL system [95]: monkeys that exerted greater effort to obtain the reward had larger volumes in the dorsolateral prefrontal cortex, centromedial amygdaloid complex, and middle cingulate cortex compared to monkeys that were only willing to exert little effort for the reward. This observation suggests that neuroanatomical variation within the MCL circuit predicts differences in the valuation of a food-based rewards, and that this association could extend to the valuation of drug rewards.

##### Impulsivity

In rodents, impulsivity is commonly defined as one of two forms: impulsive action, measured by premature responses in operant tasks such as the Five-Choice Serial Reaction Time task (5-CSRTT)); and impulsive choice, assessed by preference for a small, immediate reward over a larger, delayed reward (i.e., delay discounting). Although assessed through distinct paradigms used, impulsive action and impulsive choice are often correlated [96], and both are strong predictors of drug self-administration and behaviors related to drug reward in rodents. For example, rats that exhibit more premature, inappropriate responses in the 5-CSRTT self-administer greater amounts of cocaine [97], show a higher likelihood of developing compulsive-like cocaine-taking behaviors [37], and are more likely to resume self-administration following extinction [98] compared to rats with fewer premature responses. Similar observations have been made using delay discounting procedures [99, 100] and a gambling task [101].

##### Decision making

Variations in decision-making functions before exposure to drugs also predict subsequent drug self-administration in animals. Among rats trained to choose between a small, safe food reward and a large food reward associated with risk of mild footshock, those that select the larger, risky choice more frequently also self-administer more cocaine [102]. There are similar relationships between adaptive decision making and drug self-administration [103]. Rats that have poor reward-guided decision-making self-administer greater amounts of stimulants [82, 104]. Reward-guided decision-making functions, which emerge during adolescence [105], are predictive of cocaine self-administration in adulthood [106]. Thus, variations in decision making, including those related to developmental stage, may be important indicators of susceptibility to StUD.

#### Summary

The findings reviewed support involvement of the MCL system in both the initiation of drug use and the progression to compulsive use. Dopaminergic signaling within this circuitry modulates the rewarding effects of psychostimulants at both neural and behavioral levels. However, individual differences in dopamine function are complex and cannot be attributed solely to hyper- or hypodopaminergic states in any individual brain region. Further research—particularly studies that integrate both in vivo and ex vivo methodologies—is essential to more precisely characterize the neurobiological mechanisms underlying StUD.

Neuroimaging findings implicate dysfunction in descending prefrontal-striatal projections as a potential risk factor for addictions, including StUD. This view aligns with evidence linking impaired prefrontal-striatal connectivity to behavioral traits, such as novelty seeking and impulsivity, which are predictive of drug use in humans and self-administration in animals. Variations in this circuitry may be shaped by genetic mechanisms that have yet to be fully elucidated. In sum, current evidence suggests that key risk factors for StUD include disruptions in both ascending dopaminergic pathways and to the cortical control systems that modulate MCL dopamine activity.

## Chronic stimulant use or exposure

### Human studies

#### Neurochemistry (Fig. 1; also see [107] for table of molecular imaging findings in human subjects with SUDs)

##### Dopamine

People with StUD exhibit multiple dopaminergic deficits including deficits in dopamine transporter (DAT) availability, vesicular monoamine transporter-2 (VMAT2) levels, and stimulant-induced striatal dopamine release [108, 109]. These deficits are coupled with robust MCL dopamine responses to drug-related cues [61, 110–112].

Although relatively few studies have assessed VMAT2 and DAT, a larger body of research indicates that striatal D2/3R availability is reduced in most individuals with stimulant use disorders (StUD) involving cocaine or methamphetamine [109]. This reduction could be due to fewer receptors, decreased receptor affinity, receptor internalization, or higher levels of endogenous dopamine competing with the radiotracer. To investigate whether elevated endogenous dopamine levels were responsible, researchers conducted a study using alpha-methyl-para-tyrosine at a dose expected to significantly lower dopamine levels (and thereby increase D2/3R availability) [113]. The resulting increase in D2/3R availability was smaller in cocaine-dependent individuals compared to controls, suggesting that the reduced D2/3R availability in cocaine dependence is not due to higher extracellular dopamine.

Given the distinct functional roles of dopamine D2-type receptor subtypes [114], it is important to acknowledge that PET imaging lacks the resolution to differentiate receptors based on their cellular localization. Moreover, commonly used radiotracers do not reliably distinguish between D2 and D3 receptor subtypes (D2Rs, D3Rs). Studies using more selective radioligands have shown that chronic stimulant exposure upregulates D3 receptors (D3Rs). Postmortem analyses revealed elevated D3R levels in the ventral striatum and substantia nigra of individuals who died from cocaine overdose compared to controls [115]. Subsequent PET studies found increased D3R availability in the substantia nigra of stimulant-dependent individuals, which was associated with greater impulsivity and risky decision-making [116]. These findings suggest that D3-selective drugs may be beneficial for addressing inhibitory control deficits in StUD [117].

Cocaine- and methamphetamine-dependent participants, however, exhibit no abnormalities in striatal D1Rs [118, 119]. It has been theorized that striatal D1Rs in the “direct pathway” are involved in preparing a set of possible appropriate responses whereas striatal D2Rs in the “indirect pathway” shape and select the response [120]. Thus, D2R-expressing pathways would function to inhibit or modulate behavioral responses to phasic dopamine surges.

Presynaptic dopaminergic deficits seen in StUD may differ depending on the stimulants used. For example, individuals with a history of amphetamine misuse—but not cocaine use—exhibit reduced DAT availability [108]. Additional evidence of impaired presynaptic dopamine function in StUD includes blunted increases in extracellular dopamine in response to methylphenidate or amphetamine challenge, as well as lower brain uptake of 3,4-dihydroxy-6-[(18)F]-fluoro-l-phenylalanine ([F-18]DOPA), as a marker of dopamine synthesis capacity, compared to healthy controls [108].

##### Glutamate

Methamphetamine dependence has been associated with disruptions in glutamatergic transmission. Magnetic resonance spectroscopy studies have identified below-control levels of glutamatergic compounds (glutamate + glutamine, Glx) across multiple brain regions during early abstinence. Specifically, Glx concentrations were below control levels in the anterior mid-cingulate cortex within the first two months of abstinence [121], and in the posterior cingulate, precuneus, and right inferior frontal gyrus (IFG) within the first week (4–7 days) of abstinence [122]. Moreover, Glx levels in the posterior cingulate cortex were inversely associated with the duration of methamphetamine use, while lower right IFG Glx was correlated with greater severity of depressive symptoms. Additional deficits occurred in the insula, where lower Glx levels similarly correlated with depressive symptomatology [123]. These findings suggest a downregulation of glutamate-glutamine cycling in early abstinence, potentially representing a compensatory response to prior glutamatergic overactivity and contributing to mood dysregulation during withdrawal.

PET imaging studies have primarily focused on mGlu5, which is highly expressed throughout the MCL system and has emerged as a potential therapeutic target for StUD [124, 125]. In humans, below-control mGlu5 availability has been observed in cocaine-dependent individuals during early abstinence (e.g., 1–2 weeks) [126, 127]. In contrast, individuals abstinent from chronic methamphetamine use for approximately 42 days showed no significant global or regional differences in mGlu5 total volume of distribution compared to controls, possibly indicating receptor recovery with prolonged abstinence [128]. This interpretation is supported by preclinical evidence: in rats, mGlu5 expression in the nucleus accumbens returned to baseline levels two months after cessation of cocaine self-administration [129].

#### Morphometry/connectivity

Individuals with StUD exhibit gray-matter structural abnormalities compared to controls, particularly in frontal brain regions. These deficits include smaller volumes in the prefrontal cortex (PFC) and striatum [130–134] as well as lower OFC gray-matter tissue densities and concentrations in the orbitofrontal, cingulate and other cortical areas [135, 136]. Cortical thinning has also been observed in multiple regions, including the anterior cingulate cortex (ACC) [137–139], with reductions in cortical thickness and hippocampal volume linked to impaired memory function [133, 140], and ACC thinning linked to depressive symptoms [141]. Additionally, both functional and structural alterations have been observed in the amygdala of stimulant-dependent individuals [142, 143].

However, not all studies have found gray-matter deficits; some have shown no significant differences or even cortical or striatal enlargement [144, 145], which may reflect variability in the duration of abstinence. Notably, a longitudinal study demonstrated increases in cortical gray-matter―including the inferior frontal, angular, and superior temporal gyri, as well as the precuneus, insula, and occipital pole―after one month of methamphetamine abstinence [131]. Similarly, a cross-sectional study found that volumes of the OFC, parietal cortex and hippocampus of males increased with longer abstinence durations, up to 2.5 years, in males [146].

White-matter abnormalities, including lesions, agenesis and tissue loss, also have been observed in individuals with StUD as compared with healthy controls. In particular, subjects who used cocaine showed an elevated risk of severe hyperintense lesions in cerebral and insular white matter [147], as well as lower frontal cortical fractional anisotropy (FA) in the frontal cortex―indicative of compromised white-matter integrity and diminished OFC connectivity [148]. Additionally, unlike healthy control males aged 19–37, those who used cocaine did not exhibit the expected positive correlation between frontal and temporal white-matter volume and age, suggesting a disruption in normal white-matter maturation [149]. Chronic cocaine use also has been associated with globally lower FA and higher mean diffusivity and radial diffusion, consistent with demyelination and reduced axonal packing density [150, 151]. These white-matter deficits have been linked to heightened drug craving [150].

Individuals recently abstinent from chronic methamphetamine use have shown evidence of white-matter hypertrophy, potentially reflecting aberrant myelination or compensatory adaptations to neuronal damage [133]. Despite this hypertrophy, diffusion tensor imaging (DTI) revealed an FA deficit in prefrontal white matter, corpus callosum and the corona radiata. Notably, FA deficits in the midcaudal superior corona radiata correlated with depressive and generalized psychiatric symptoms [152]. Chronic use of either cocaine or methamphetamine has been linked to lower FA and higher radial diffusivity across association, commissural, and projection white-matter tracts; methamphetamine use, in particular, has also been associated with lower axial diffusivity [153]. More recently, the volume of white matter lesion―measured via hypointensities on T1-weighted MRI scans―was higher in participants who had used methamphetamine compared to controls [154]. The volume of white matter hypointensities was larger for older participants across control and methamphetamine groups but larger for the methamphetamine group irrespective of age, indicating an early age-related change [154].

#### Behavioral deficits: links to MCL neurochemical and functional abnormalities

Individuals with StUD perform below control levels on cognitive tasks, particularly tasks that involve behavioral inhibition or cognitive control. Those with cocaine use disorder exhibit deficits in executive functions, including focusing of attention, working memory and response inhibition [155]. Similarly, methamphetamine-dependent subjects underperform healthy controls in several functions [156], as indicated on tests of motor response inhibition, cognitive flexibility [157–161], and emotion regulation [162]. However, the ecological validity of these laboratory measures for predicting real-world drug use behavior remains uncertain.

These cognitive weaknesses accompany functional abnormalities related to cognitive control, as measured with fMRI. During the incongruent condition of the Stroop task, methamphetamine-dependent subjects have below-control cortical activation in multiple cortical areas important for executive functions―right inferior frontal gyrus, supplementary motor cortex, anterior cingulate gyrus, anterior insula [163, 164]―suggesting that hypofunction in MCL cortical areas may underlie their cognitive control deficits. Indeed, neural functional connectivity during a working memory task is attenuated in methamphetamine-dependent subjects compared to controls and proposed to represent a decoupling in network dynamics that occurs during early abstinence from methamphetamine use [165].

The MCL system also has been implicated in maladaptive decision making by individuals with StUD, as evidenced by findings from temporal (delay) discounting tasks [166, 167] and experimental paradigms examining reward uncertainty and risk processing [168–171]. For example, discounting rates were negatively correlated with striatal D2/3R availability in methamphetamine-dependent individuals, but not in controls [166]. Functional differences were also observed in frontoparietal regions [167]; whereas control participants showed less recruitment during decisions involving large differences between small, immediate and larger delayed rewards, methamphetamine-dependent individuals did not, suggesting inefficient cortical processing during value-based decision making.

Risky decision making has been evaluated in tests that involve adapting behavior based on feedback during the task. One example is the Balloon Analogue Risk Task (BART) [172], in which successive trials present increasing stakes (greater risk of loss, greater potential reward). By using a computational model, behavior on the task was deconstructed into risk-taking and behavioral updating [171]. Methamphetamine-dependent participants underperformed controls and exhibited slower behavioral updating, which was positively correlated with D2/3R availability in the striatum and globus pallidus. The results suggested that dysregulation of D2/3R signaling contributes to the decision-making deficit.

Maladaptive decision making by methamphetamine-dependent subjects may reflect circuit-level dysfunction, as indicated in an fMRI study [173]. Methamphetamine-dependent subjects exhibited heightened resting-state connectivity within the MCL, coupled with weaker prefrontal cortical connectivity as compared with controls. In addition, parametric modulation of activation by the stakes (magnitude of potential reward and loss in a trial) was stronger in the ventral striatum but weaker in the right dorsolateral PFC in methamphetamine-dependent participants than control individuals. The findings are consistent with a pattern that may create a bias toward reward-driven behavior over cognitive control in methamphetamine use disorder.

#### Summary

Neuroimaging studies have consistently identified neurochemical, structural, and functional abnormalities within the mesocorticolimbic (MCL) circuitry of individuals with chronic psychostimulant use. These neurochemical targets, which have been implicated in behavioral deficits linked to StUD, may thereby modulate susceptibility to relapse. Dysregulation of both pre- and postsynaptic dopaminergic markers has been observed, with reductions in striatal D2/3 receptor (D2/3R) availability—rather than D1R—linked to elevated trait impulsivity and maladaptive decision-making. However, the relationship between dopaminergic dysfunction and the progression from initial to chronic use remains unresolved; at-risk individuals may present with either hypo- or hyperdopaminergic profiles across both presynaptic and postsynaptic domains (see above, vulnerability section). Despite this heterogeneity, converging evidence in StUD supports the therapeutic potential of selectively enhancing D2/3R over D1R signaling, or modulating D2R versus D3R activity, particularly in the context of impaired inhibitory control. Additionally, targeting glutamatergic transmission may offer a complementary strategy for ameliorating the cognitive deficits observed in StUD. Collectively, these neurochemical substrates—implicated in both the expression and persistence of behavioral impairments—represent promising targets for interventions aimed at reducing relapse vulnerability.

Gray- and white-matter abnormalities across cortical and subcortical nodes of the MCL system have been documented in individuals with StUD and their unaffected siblings, suggesting a potential heritable liability. These structural alterations, which are associated with cognitive and behavioral impairments in chronic users, may inform the development of anatomically targeted interventions, including neuromodulatory approaches (see below).

### Animal studies

#### Neurochemistry (Fig. 1)

##### Dopamine

A large body of preclinical evidence indicates that chronic use of stimulants disrupts dopamine function, consistent with early reports in post-mortem autoradiography studies [174]. NHPs that self-administered cocaine for ~20 months had lower striatal D2/3R availability compared to controls. Subsequent PET studies in NHPs, where measurements can be repeated within the same subject, have found that extensive exposure to stimulant drug regimens decreases striatal D2/3R availability [175, 176]. These effects appear, in part, to be sensitive to patterns of drug exposure (stable, escalation, and/or binge-like), and the time when post-drug measurements are collected, suggesting that decrements in D2/3R availability likely reflect adaptive responses to receptor stimulation [176]. Indeed, microdialysis and fast-scan cyclic voltammetry found marked reductions in striatal dopamine tone following drug self-administration [177, 178] that may, in part, recover during extended periods when drug is unavailable.

The cited PET studies did not distinguish between D2 and D3 receptor subtypes, which may have opposing roles in stimulant self-administration [179]. Thus, reductions in D2/3R receptor availability following stimulant exposure may reflect different and potentially opposite changes in D2R and D3R signaling. Of note, cocaine exposure decreased striatal D2R mRNA in rats [180].

Research on stimulant addiction has predominantly focused on D2/3Rs, with comparatively limited investigation into the role of D1 receptors (D1Rs) in animal models. Longitudinal studies examining D1R dynamics in response to stimulant administration are particularly scarce. However, ex vivo autoradiographic studies have demonstrated reduced striatal D1R binding in NHPs following cocaine self-administration [174], with levels normalizing by 90 days after removal of access to cocaine [181]. Yet, D1R receptor signaling is necessary for cocaine self-administration [182] and has been implicated in the rewarding effects of stimulants. Moreover, the deficit in striatal D2/3R but not D1R availabilities in human subjects with StUD (see above) supports a model whereby D1R-D2R signaling dysregulation, rather than isolated receptor changes, underlies chronic stimulant use, as suggested by the response to alcohol in mice [183].

In addition to these post-synaptic changes, presynaptic dopaminergic markers can change with stimulant exposure, although these effects vary with the drugs. For example, PET studies have found that DAT availability is not altered following cocaine self-administration [72, 184] but is decreased following an escalating dose of regimen of methamphetamine in NHPs [175] and rats [185]. The methamphetamine-induced reduction in DAT occurs without toxicity to or loss of monoaminergic neurons, suggesting that changes in DAT are likely in adaptation to extreme elevations in synaptic dopamine levels that follow methamphetamine exposure.

The effects of stimulant use on VMAT2 are less clear. Despite some evidence for lower VMAT2 in cocaine-dependent individuals [108], studies in animals have not found consistent evidence that stimulant self-administration reduces VMAT2. For example, VMAT2 binding in cocaine-exposed rats was similar to or above values in drug-naïve rats [186, 187] whereas PET studies in NHPs have shown a robust decrease in VMAT2 following cocaine self-administration [188]. These inconsistencies may reflect differences in the species (rat vs. monkey), or in the techniques or ligands used to quantify VMAT2 binding (PET vs. postmortem),or in the drug exposure regimens. Nevertheless, longitudinal studies of stimulant effects on VMAT2 warrant further investigation.

##### Glutamate

Studies in animals found dynamic changes in glutamate transmission following stimulant exposure (for review, see [124]). For example, PFC and striatal glutamate levels are lower in rats during stimulant self-administration [189, 190]. During withdrawal, however, glutamate levels appear to normalize in the PFC but remain attenuated in the nucleus accumbens [84]. Additionally, observations that glutamate levels are greater than in control animals during drug- and cue-induced reinstatement tests [191, 192] suggest a role of glutamate in drug-seeking behaviors. Although these changes in glutamate levels appear to be transient, dysregulation of glutamate receptors persists. Expression of mGlu5 protein is lower in the nucleus accumbens of rats following short (<1 day) and extended (>60 days) periods of withdrawal from cocaine [193–195]. In other brain regions, stimulant-dependent changes in mGlu5 appear to be dynamic and sensitive to the environment. PET studies have found decreases in mGlu5 availability the hippocampus of rats two weeks after removal of access to cocaine [84], but increases in the PFC of rats that underwent extinction training during the withdrawal period [85]. In extrastriatal regions, mGlu5 signaling, therefore, may be necessary for extinction of cocaine-associated cues [196] and regulation of relapse-like behaviors.

Signaling through the mGlu2/3 receptor may govern reward processing and drug seeking by promoting dopamine and glutamate release [197]. Levels of mGlu2/3 receptors are lower in striatal and cortical regions of rats following extended methamphetamine self-administration [198], and mGlu2/3 receptor agonists appear to block reinstatement of drug-seeking behaviors in rats [199–201]. Therefore, restoring mGlu2/3 signaling when access to drug is removed may reduce relapse to drug-seeking behaviors.

#### Morphometry/connectivity/neuroimaging

Many of the morphometric abnormalities observed in stimulant-dependent individuals using MRI have been recapitulated in animal studies. Gray-matter density is reduced in the OFC, insula, amygdala, and temporal cortex of NHPs that self-administered cocaine for 12 months [202], with similar effects in the prefrontal cortex of methamphetamine-exposed NHPs [203]. Similar disruptions in structural and functional connectivity occur in rats following exposure to stimulants [204–206].

Recent advances employing viral-genetic tools and optical recording methods [207] have begun to elucidate MCL network dynamics with unprecedented cell-type specificity and temporal resolution. Fiber photometry studies of midbrain projections to the nucleus accumbens reveal that fluctuations in mesolimbic reward-related signaling can predict future alcohol intake [208] and reinstatement of alcohol-seeking behaviors in rodent models [209]. Similarly, dopamine release in the nucleus accumbens during cocaine self-administration has been shown to predict reinstatement of cocaine-seeking behavior [210], implicating drug-induced dysregulation of mesolimbic dopamine transmission in the persistence of drug-seeking behaviors in StUD.

Future studies that expand these circuit-level investigations to other limbic and cortical regions before and after stimulant self-administration will be critical to enhancing our understanding of MCL disruptions in StUD. Moreover, integrating these mechanistic approaches with traditional neuroimaging measures could help narrow in on specific circuits, cell types, and signaling mechanisms that are altered by stimulant use.

#### Behavior

##### Sensitivity to reward

The impact of stimulant exposure on reward sensitivity remains unclear, in part due to the conceptual ambiguity surrounding the construct itself [211, 212], and also because patterns and duration of drug exposure can have bidirectional effects on reward processing. For example, chronic administration of stimulants can increase intracranial self-stimulation reward thresholds [213, 214] and attenuate the response of dopamine neurons to cocaine [177], suggesting that extensive stimulant exposure leads to blunting of biobehavioral responses to rewards. In contrast, intermittent and/or acute exposure to stimulants enhances—or sensitizes—the biobehavioral response to rewards [215–217], potentially serving as the mechanism through which pathological levels of drug ‘wanting’ develop during addiction [218]. These different effects of stimulants on reward sensitivity may not involve mutually exclusive processes but rather may reflect the underlying dynamics of addiction pathology that emerges in response to changing patterns of drug self-administration.

##### Impulsivity

Both impulsive action and impulsive choice are predictive of stimulant self-administration in animal model; however, the long-term effects of stimulant exposure appear to impact these domains differently. Premature responding of rats in the 5CSRT task—a measure of impulsive action*—*shows only transient elevation during initial periods of cocaine self-administration, returning to baseline with continued exposure, potentially reflecting the development of tolerance [219, 220]. In contrast, stimulant exposure produces a robust and persistent shift toward impulsive choice, characterized by selection of smaller, immediate rewards over larger, delayed alternatives (for review, see [221]). This dissociation between the persistent effects of stimulants on impulsive choice versus the transient effects on impulsive action has yet to investigated systematically in humans.

##### Decision making

Stimulant exposure disrupts decision making under risk, but this effect seems to be age-dependent. In rodent models, cocaine exposure during late adolescence or early adulthood increases risky choice, whereas similar exposure in late adulthood has no such [222]. Although the underlying mechanisms remain unclear, developmental cocaine exposure may interfere with maturation of dopaminergic innervation of the prefrontal cortex―a process that continues through late adolescence into early adulthood [223].

Chronic stimulant exposure also impairs cognitive flexibility, particularly in tasks requiring adaptation to changing contingencies, environments, or outcomes. Both NHPs and rodents with a history of chronic stimulant exposure show deficits in reversal-learning tasks, continuing to select previously reinforced options despite shifts in reinforcement contingencies [224, 225]. This increase in perseverative responding has been linked to impaired use negative feedback to guide behavior [85, 224], and may contribute to compulsive drug-seeking and -taking patterns characteristic of StUD.

## Conclusions

Understanding the neural processes involved in all stages of drug use is essential to guide efforts to mitigate the adverse consequences of StUD. These include susceptibility to using drugs, transitions from use to dependence, and drug-induced impairments after long-term use. Human and animal studies have implicated the MCL in each stage. Sibling and family studies suggest that distinct MCL features confer vulnerability vs resiliance to StUD. Behavioral characteristics such as impulsivity and deficits in inhibitory control—both associated with MCL dopaminergic function—are linked to increased vulnerability to SUDs in humans and a greater likelihood of animals to self-administer drugs, including stimulants. After drug use, there are both behavioral impairments and deficits in MCL function, both of which can worsen the process of recovery (see Fig. 1 table summarizing MCL dopaminergic and glutamatergic mechanisms associated with risk for SUD and those mechanisms affected by chronic stimulant exposure).

A major goal of uncovering the brain mechanisms of StUD vulnerability is to identify individuals at risk and develop preventive measures. Longitudinal studies beginning prior to drug exposure are necessary to determine the extent to which findings observed following chronic stimulant misuse contribute to the development of StUD. This need has been a driving force of longitudinal studies, such as the ABCD Study® [226], directed at identifying psychosocial factors that predict early substance use, as well as neural underpinnings, including MCL involvement, in vulnerability (e.g., [227]). Although behavioral measures can help to predict StUD, a deeper knowledge of the neural processes that control the behaviors is essential to develop novel prevention and treatment approaches. Such knowledge can only be acquired through translational research using both humans and laboratory animal models that provide more mechanistic insights.

We have reviewed evidence that both humans with StUD as well as animals chronically treated with stimulant drugs exhibit deficits at the biochemical, neural, and behavioral levels. Although behavioral and neural differences between people who do or do not use drugs may reflect either pre-existing differences or the consequences of drug use, understanding how they arise will likely aid in the development of novel treatment strategies for StUD. Causal factors can be investigated in human longitudinal studies, and in preclinical studies that provide control over drug exposure. They can also be studied by careful comparison of risk factors and impairments seen in those who engage in long-term use. It must be acknowledged, however, that individuals with StUD often use other drugs that can hijack the MCL system, and that effects of stimulants alone can be exacerbated by polydrug use.

To date, pharmacological interventions for stimulant use disorder (StUD) have yielded limited success, with the most robust evidence supporting psychosocial approaches such as contingency management [228]. Advances in the neurobiology of MCL function in the context of stimulant exposure may inform the development of targeted pharmacotherapies that can be deployed alongside behavioral interventions. Given the heterogeneity of dopaminergic alterations across receptor subtypes following chronic stimulant use, agents with greater specificity may be required to address the persistent cognitive impairments characteristic of StUD. Furthermore, the glutamatergic system remains an underexplored avenue for drug development, including the potential of allosteric modulators targeting glutamate receptors. Beyond pharmacological strategies, novel neuromodulatory interventions are gaining traction. Techniques such as transcranial magnetic stimulation—already FDA-approved for major depressive disorder, obcessive-compulsive disorder, migraine headaches and smoking cessation—represent a promising therapeutic modality for StUD and other SUDs [229]. Emerging technologies, including low-intensity focused ultrasound, offer unprecedented spatial resolution and may enable modulation of both cortical and subcortical MCL components implicated in addiction pathology [230]. These developments underscore the critical importance of delineating the circuit-level disruptions within the MCL system reviewed here, to inform future therapeutic innovation.
